# Time Optimal Trajectory Planing Based on Improved Sparrow Search Algorithm

**DOI:** 10.3389/fbioe.2022.852408

**Published:** 2022-03-22

**Authors:** Xiaofeng Zhang, Fan Xiao, XiLiang Tong, Juntong Yun, Ying Liu, Ying Sun, Bo Tao, Jianyi Kong, Manman Xu, Baojia Chen

**Affiliations:** ^1^ Key Laboratory of Metallurgical Equipment and Control Technology of Ministry of Education, Wuhan University of Science and Technology, Wuhan, China; ^2^ Research Center for Biomimetic Robot and Intelligent Measurement and Control, Wuhan University of Science and Technology, Wuhan, China; ^3^ Hubei Key Laboratory of Mechanical Transmission and Manufacturing Engineering, Wuhan University of Science and Technology, Wuhan, China; ^4^ Precision Manufacturing Research Institute, Wuhan University of Science and Technology, Wuhan, China; ^5^ Hubei Key Laboratory of Hydroelectric Machinery Design & Maintenance, China Three Gorges University, Yichang, China

**Keywords:** trajectory planning, inverse kinematics, configuration space, time optimization, improved sparrow search algorithm

## Abstract

Complete trajectory planning includes path planning, inverse solution solving and trajectory optimization. In this paper, a highly smooth and time-saving approach to trajectory planning is obtained by improving the kinematic and optimization algorithms for the time-optimal trajectory planning problem. By partitioning the joint space, the paper obtains an inverse solution calculation based on the partitioning of the joint space, saving 40% of the inverse kinematics solution time. This means that a large number of computational resources can be saved in trajectory planning. In addition, an improved sparrow search algorithm (SSA) is proposed to complete the solution of the time-optimal trajectory. A Tent chaotic mapping was used to optimize the way of generating initial populations. The algorithm was further improved by combining it with an adaptive step factor. The experiments demonstrated the performance of the improved SSA. The robot’s trajectory is further optimized in time by an improved sparrow search algorithm. Experimental results show that the method can improve convergence speed and global search capability and ensure smooth trajectories.

## Introduction

Robots are one of the vehicles for replacing some of the work of humans, combining the strengths of many disciplines and being used in a wide range of fields, such as mechanism (Yoshikawa, 1985; Park, 1995), computer vision (Huang et al., 2020; Bai et al., 2022), intelligent control (Yang et al., 2019; Yu et al., 2020; Yun et al., 2022a), artificial intelligence (Yu et al., 2019; Li et al., 2019; Sun et al., 2020a), signal processing (He et al., 2019; Jiang et al., 2019; Ma et al., 2020; Sun et al., 2020b; Weng et al., 2021), etc.. Time is the primary factor to be considered in engineering scenarios, and optimizing time is of great research significance for improving the efficiency of robotic arms. Time optimization aims to maximize the execution efficiency of the motor within the permissible limits. Simply increasing the torque and power of the motor is not feasible, as it results in greater energy consumption and inertia. Depending on the space in which the planning object is located, it can be divided into trajectory planning in Cartesian space and trajectory planning in joint space. When planning trajectories in Cartesian space, the position and pose of the robot arm end-effector (EE) are intuitive (Kant and Zucker, 1986; Xu et al., 2009; Zheng et al., 2009), making it more suitable for scenarios with strict requirements on the trajectory movement process, such as machining (Sun et al., 2020c), welding (Hao and Wang, 2021), rehabilitation robot (Liu et al., 2016; Sun et al., 2018; Li et al., 2020; Luo et al., 2020), etc. However, inverse kinematic calculation takes up significant computational resources during the trajectory motion, and it is difficult to circumvent the kinematic singularity problem. When planning a trajectory in joint space (Chen and Zalzala 1997; Verscheure et al., 2009; Stilman, 2010), the object of planning is the joint angles corresponding to the target position, the whole process is done in joint space, and the motion is fully accessible, avoiding a large number of inverse kinematic calculations and eliminating the need to consider the problem of singularities, thus making it faster. After completing the planning in joint space, the trajectory needs to be mapped back to Cartesian space. The mapping process is highly non-linear, so the trajectory is unpredictable without constraints (Liu et al., 2013). However, many scenarios only require identifying several key points on the trajectory and do not require tracking the movement between these points, such as rigid body grasping (Miao et al., 2015; Hu et al., 2019; Jiang and Zheng, 2019; Duan and Sun, 2021; Jiang et al., 2021; Liu et al., 2022a), intelligent sorting, plant planting, etc. From a control point of view, the motor drive system acts directly on the joint angles of the axes so that the smoothness of the joint trajectory is more important than the smoothness of the trajectory in task space.

Time optimization is a single objective optimization problem for trajectory planning. The minimum time for the trajectory motion is calculated with the constraint that the velocity and acceleration do not exceed a set maximum value, provided that the trajectory and velocity are smoothly continuous and the acceleration is continuous (Gasparetto et al., 2012). The optimal time problem is generally solved by determining the interpolation function of the trajectory and then determining the coefficients of the interpolation function to ensure time is optimal. Polynomials are widely used in the choice of trajectory interpolation functions (Biagiotti and Melchiorri, 2008). The polynomials’ order grows larger to obtain more accurate trajectories, which causes the Runge’s phenomenon. Multi-segment polynomials are used in most scenarios in order to eliminate this effect. However, this brings about the creation of non-differentiable points within the interpolation function, which has a negative effect on the normal operation of the motor. The polynomial is at least third order derivable to ensure smoothness of velocity and continuity of acceleration. Higher orders provide more parameters and mean that the calculation becomes difficult. Alternatively, B splines have excellent performance in smoothing, and Thompson SE developed a method for constructing joint trajectories using B splines (Thompson and Patel, 1987). However, the properties of B splines dictate that the trajectories are fitted and not interpolated, and the absolute accuracy of the interpolated points cannot be guaranteed.

This work aims to reduce the time of robot trajectory planning in terms of both inverse solution calculation and trajectory optimization. The algorithm used for trajectory optimization can be extended to other configurations of robots. This paper completes the trajectory planning of a UR5 collaborative robot (cobot) in joint space by means of cubic spline interpolation and obtains a time-optimal trajectory using an improved sparrow search algorithm (SSA). The complete flow of the trajectory motion is considered in this paper for the time optimization of the trajectory and the optimization of the inverse solution calculation method. The method of partitioning the joint space of the UR5 cobot is discussed, and one-to-one correspondence with the subspaces is obtained, significantly reducing the time required for the inverse kinematics (IK) calculation. The smoothness of the joint trajectory and the velocity is ensured by a cubic spline interpolation method. The problem of its tendency to fall into local optima is improved by improving the sparrow search algorithm to obtain a time-optimal trajectory with maximum velocity and acceleration as constraints.

This paper is organized as follows. Section 2 details other studies relevant to the work in this paper. The mathematical model for time-optimal trajectory planning is presented in Section 3, and the problems and solutions in the various processes of trajectory planning are discussed in detail. The sparrow search algorithm and its improved methods are shown in detail in Section 4. Section 5 shows the experimental results of the algorithm. Some outlooks and conclusions are given in Section 6.

## Related Works

The time-optimal problem of trajectories has been actively studied. Earlier proposed algorithms (Bobrow et al., 1985; Shin and McKay, 1985) are based on the position-phase plane. These algorithm’s transform the time-optimal problem into a function of θ and v as parameters to find the optimal problem. In short, for each point of the path, the minimum time to pass the entire path is obtained by passing at the maximum speed. The maximum velocity allowed for each point is found in the plane formed by *θ* and *v*, and is made continuous when switching from point to point. These methods do not consider the acceleration continuity, which is not possible for an actuator moving in actual operation to produce discontinuous acceleration. This approach of ignoring actuator dynamics leads to two adverse effects: first, the discontinuous acceleration causes the actuator motion always to be delayed concerning the reference trajectory. This significantly reduces the tracking accuracy of the trajectory. In addition, constant switching can achieve discontinuous acceleration, but this introduces high-frequency oscillations to the actuator.

Solutions to these problems may be found in this literature (Shiller, 1996; Constantinescu, 1998; Constantinescu and Croft, 2000). In these methods higher order derivatives are added for finite control, which requires the establishment of third order dynamic equations. However, building accurate kinetic models is often challenging to accomplish.

Another approach that does not require a dynamics model is to use a smoothing function to express the trajectory in joint space. The torque of the actuator is directly reflected in the joint variation of the robot so that a smooth trajectory will result in a smooth model. In the usual case, the spline interpolation function is widely used. Three constraints need to be considered after describing the joint trajectory using the spline function.

1) Speed limit;
2) Acceleration limit;
3) Jerk limit.

The third spline function has time as the horizontal axis and joints as the vertical axis, and the third-order derivability ensures that the acceleration is continuous. The calculation of the optimal trajectory time is completed by finding the minimum value of time under the satisfied constraints.

Tandem robots are generally made up of multiple joints, and the motion of each joint is coupled, making it challenging to complete the optimization process through numerical solutions. Swarm intelligence search algorithms have shown great vitality in such problems (Tian et al., 2020; Chen et al., 2021a; Chen et al., 2021b; Chen et al., 2022). Swarm search algorithms are widely used in robotics, such as inverse solution computation (Zhao et al., 2022), control (Liu G et al., 2021; Wu et al., 2022), pose recognition (Li et al., 2019a; Tao et al., 2022a) and other nonlinear problems (Huang et al., 2019; Sun et al., 2020d; Hao et al., 2022). Recently published optimisers (Ghafori and Gharehchopogh, 2012; Abedi and Gharehchopogh, 2020; Abdollahzadeh et al., 2021a; Gharehchopogh et al., 2021a; Abdollahzadeh et al., 2021b; Benyamin et al., 2021; Gharehchopogh et al., 2021b; Gharehchopogh and Abdollahzadeh, 2021; Goldanloo and Gharechophugh, 2021; Mohammadzadeh and Gharehchopogh, 2021; Zaman and Gharehchopogh, 2021; Gharehchopogh, 2022) have achieved good performance but may not suit industrial scenarios with high real-time requirements. The particle swarm optimization algorithm (PSO) is used to search for the global time-optimal trajectory of a spatial robot in conjunction with robot dynamics (Huang and Xu, 2006). Huang uses a multi-objective particle swarm optimization algorithm optimization method for the multi-objective optimization of the motion trajectory of a space robot (Huang et al., 2008). Liu and Zhang (Zhang et al., 2018; Liu and Rodriguez, 2021) used a quintuple polynomial for trajectory planning for the PUMA560 robot and proposed an improved genetic algorithm (GA) to accomplish time-optimal trajectory planning.

These works demonstrate the feasibility of the group search algorithm for this problem, but accuracy and convergence speed remains problematic. The sparrow search algorithm is an algorithm proposed by Jiankai Xue (Xu et al., 2022) in 2020. The algorithm outperforms PSO, GA, grey wolf optimization algorithms (GWO). It is widely used other search algorithms on uni-modal and multi-modal test functions and is widely used in problems such as path planning for mobile robots (Liu et al., 2022b), control of photovoltaic microgrids (Yuan et al., 2021) and optimization of battery stack model parameters (Liu Y et al., 2022). We find that SSA is suitable for time-optimal trajectory planning problems and improves the initial population generation in the original algorithm through the Tent chaotic mapping method. An adaptive step size factor adjusts the individual update to improve the global search capability. Time-optimal trajectory planning was completed on the UR5 collaborative robot, and experimental results demonstrate the effectiveness of the method.

A complete process includes trajectory determination, inverse solution solving, and trajectory optimization in practical motion. Depending on the scenario, the conditions for determining the trajectory are different. Obtaining information about the environment in these scenarios can be done by different sensors, such as myoelectric signals (Li et al., 2019b; Cheng et al., 2020; Cheng et al., 2021; Yang et al., 2021; Liu et al., 2022c), visual sensors (Jiang and Li, 2019; Jiang and Li, 2021; Tan et al., 2020; Huang et al., 2021; Liao et al., 2021; Liu X. et al., 2022; Sun et al., 2021, 2022; Yun et al., 2022b), multi-sensor fusion (Li et al., 2019c; Liao et al., 2020; Tao et al., 2022b), etc. The theoretical time required for the robot to complete the motion of a specified trajectory includes the motor execution time and the kinematic computation time. Therefore, trajectory planning is closely integrated with inverse kinematic solving. However, none of the above methods takes into account the time taken up in the trajectory planning by the inverse solution calculation. Based on the work described above, this paper also combines the unique domain theory to improve the computational efficiency of the algorithm further when the trajectory is in motion.

## Time-Optimal Trajectory Planning

A complete trajectory planning process should include key point selection, inverse solution calculation, trajectory function setting and optimization. The coordinates of the key points on the trajectory are generally known in a Cartesian coordinate system. The inverse kinematics calculations allow the conversion of Cartesian coordinates into joint space, and the inverse kinematics of multi-joint robots is often multi-solvable. The UR5 cobot has eight sets of inverse solutions for the same pose. Assume that given a discrete sequence *p*_*i*_ of n interpolated sample points, the corresponding inverse solution of the joint angles is ***Q***_***i***_ =[***θ***_*i*1_, ***θ***_*i*2_,..., ***θ***_*i*8_] = *IK*(*p*_*i*_), where ***θ***_*ij*_ is a one-dimensional vector of six elements corresponding to the angle of each joint, *i* = 1, 2,..., *n*, *j* = 1, 2, ..., 8. It is necessary to filter the inverse solution by a certain condition after the joint angle has been found, which often follows the principle of minimum joint variation between adjacent poses. As each joint has a different influence on the EE’s trajectory, the amount of variation needs to be weighted in the calculation. The joint angle *q*_*i*_ corresponding to the *ith* sample point, i.e. the *ind-*th set of solutions in ***Q***_***i***_, can be found from the following equation

$$\begin{array}{l} \left(\Delta \theta ,ind\right)=\min\left(\sum_{m=1}^{6}w_{m}\left|\theta_{ij,m}-q_{i-1,m}\right|\right)\\ q_{i}=Q_{i}\left(ind\right) \end{array}$$ (1)

where *q*_*i*-1_ is the joint angle corresponding to the (*i*-1)-th sample point, m is the ordinal number of the joint angle, *m* = 1,2,...,6. *w*_*m*_ is the weighting factor corresponding to the *mth* joint, ∆*θ* is the minimum variation, and ind is the ordinal number of the minimum variation corresponding to in ***Q***_***i***_.

The calculation and filtering of the inverse solution can occupy a lot of computational resources and waste much time, especially when the number of sample points is large. The literature (Wenger, 1992; Wenger, 2019) analyzed in depth the method of partitioning joint spaces and obtained the conclusion that the Jacobi matrix equation (det(***J***) = 0) can partition the joint space into subspaces with the same number of solutions as the inverse, and these subspaces are called unique domains. However, they only provide a geometric analysis and do not suggest specific applications. The determinant of the Jacobi matrix for the UR5 cobot is

$$\begin{array}{l} \left|J\right|=Pn_{1}Pn_{2}Pn_{3}\\ Pn_{1}=a_{2}a_{3}s_{5}\\ Pn_{2}=s_{3}\\ Pn_{3}=a_{2}c_{2}+a_{3}c_{23}-d_{5}s_{234} \end{array}$$ (2)

where *c* represents the function cos, *s* denotes the function sin, subscript *i* denotes *θ*_*i*_, subscript *ij* denotes *θ*_*i*_+*θ*_*j*_, subscript *ijk* denotes *θ*_*i*_+*θ*_*j*_+*θ*_*k*_. The rest of the paper uses expressions simplified in this way.

Each unique domain corresponds to a unique inverse resolution. It is shown that the choice of a suitable unique domain ensures the uniqueness of the inverse solution analytic, thus avoiding the problem of multiple solutions to the inverse kinematics. The pose ***T*** of EE is already known in inverse kinematic computing, and the general form of ***T*** can be expressed as follows

$$T=\left[\begin{array}{l} \vec{n}\;\vec{o}\;\vec{a}\;\vec{p}\;\\ 0\;0\;0\;1 \end{array}\right]\;\;=\left[\begin{array}{cccc} n_{x} & o_{x} & a_{x} & p_{x}\\ n_{y} & o_{y} & a_{y} & p_{y}\\ n_{z} & o_{z} & a_{z} & p_{z}\\ 0 & 0 & 0 & 1 \end{array}\right]$$ (3)

where $\vec{n}$ is the normal lapse, $\vec{o}$ is the direction vector, $\vec{a}$ is the approach vector, and $\vec{p}$ is the position vector.

The unique domain corresponding to the inverse solution of the trajectory does not change when the robot’s configuration does not change throughout its motion. Therefore only one unique domain corresponding to the inverse solution can be used when doing trajectory planning. The inverse solution has only one set of analytic solutions when the unique domain is determined (Liu Y et al., 2022). Combined with the method proposed by Xiao (Xiao et al., 2021), the calculation method of inverse kinematics of UR5 cobot is obtained. The IK analytical expression is

$$\begin{array}{l} \theta_{1}=\mathrm{atan}2\left(\frac{-d_{4}p_{x}+Ap_{y}}{-d_{4}^{2}-A^{2}},\frac{d_{4}p_{y}+Ap_{x}}{-d_{4}^{2}-A^{2}}\right)\\ \theta_{2}=\mathrm{atan}2\left(\frac{-MP_{2}-NP_{2}}{-P_{1}^{2}-P_{2}^{2}},\frac{-MP_{2}+MP_{1}}{-P_{1}^{2}-P_{2}^{2}}\right)\\ \theta_{3}=\mathrm{atan}2\left(s_{3},c_{3}\right)\\ \theta_{4}=\;\mathrm{atan}2\left(o_{z}s_{5}+c_{5}\left(n_{z}c_{6}-a_{z}s_{6}\right),a_{z}c_{6}+n_{z}s_{6}\right)-\theta_{2}-\theta_{3}\\ \theta_{5}=\mathrm{atan}2\left(k_{2}\sqrt{1-{\left(o_{y}c_{1}-o_{x}s_{1}\right)}^{2}},o_{y}c_{1}-o_{x}s_{1}\right)\\ \theta_{6}=\mathrm{atan}2\left(\left(n_{x}s_{1}-n_{y}c_{1}\right)/s_{5},\left(a_{x}s_{1}-a_{y}c_{1}\right)/s_{5}\right) \end{array}$$ (4)

where

$$\begin{array}{l} A=k_{1}\sqrt{p_{x}^{2}+p_{y}^{2}-d_{4}^{2}}\\ c_{3}=N^{2}+M^{2}-a_{2}^{2}-a_{3}^{2}/2a_{2}a_{3},\;s_{3}=k_{3}\sqrt{1-c_{3}^{2}}\\ N=d_{1}-p_{z}-d_{5}c_{234},\;M=k_{3}\sqrt{p_{x}^{2}+p_{y}^{2}-d_{4}^{2}}+d_{5}s_{234}\\ P_{1}=a_{3}s_{3},\;P_{2}=a_{2}+a_{3}c_{3} \end{array}$$ (5)

and *a*_2_, *a*_3_, *d*_4_, *d*_5_ are the DH parameters of the robot and the exact values can be obtained in Table 1. *k*_1_, *k*_2_ and *k*_3_ as 1 or −1.

**TABLE 1:** DH parameters of UR5 cobot, including link offset *d*_*i*_, link length *a*_*i*_, twist angle *α*_*i*_ and joint angle *θ*_*i*_ *.*

No	*d*_*i*_ (m)	*a*_*i*_ (m)	*α*_*i*_ (rad)	*θ*_*i*_ (rad)
1	0.1625	0	π/2	*θ*_1_
2	0	-0.425	0	*θ*_2_
3	0	-0.3922	0	*θ*_3_
4	0.1333	0	π/2	*θ*_4_
5	0.0997	0	-π/2	*θ*_5_
6	0.0996	0	0	*θ*_6_

The inverse solution calculation leads to values of *k*_1_, *k*_2_, and *k*_3_ that are not unique, which is why the joints have multiple solutions. There are eight sets of solutions in total. As the robot’s configuration does not change when the trajectory is in motion, it is not necessary to consider all cases of *k*_1_, *k*_2_, *k*_3_, and only a set of values needs to be chosen. According to the unique domain theory, we let *k*_1_ =1, *k*_2_= −1, *k*_3_= 1.

The inverse kinematic solution of the UR5 robot can be completed from Eqs. 4 and 5. The trajectory between sample points is generally done by interpolation. Assuming that the time corresponding to sample point *p*_*j*_ is *t*_*i*_, the whole trajectory *s*(*t*) can be expressed as

$$\begin{array}{l} s\left(t\right)=\{\begin{array}{c} s_{1}\left(t\right),t\in \left[t_{0},t_{1}\right]\\ s_{2}\left(t\right),t\in \left[t_{1},t_{2}\right]\\ \vdots \\ s_{n}\left(t\right),t\in \left[t_{n-1},t_{n}\right] \end{array}\\ s_{j}\left(t\right)=u_{k,j}t^{k}+u_{k-1,j}t^{k-1}+\ldots +u_{1,j}t+u_{0,j} \end{array}$$ (6)

where *u*_*k*,*j*_, *u*_*k*-1,*j*_, ..., *u*_0,*j*_ are constant coefficients and *k* is the order of *s*_*j*_(*t*), *j* = 1, 2, ..., *n*.

A spline curve is a special function defined by a polynomial segment. In engineering applications, spline interpolation is more reliable than polynomials. The interpolation error is small even if the order of the spline curve is not high, thus avoiding Runge’s phenomenon. In order to balance the smoothness of the trajectory with the speed of the calculation, we use cubic splines, which guarantee the continuity of the acceleration. Thus the trajectory *s*_*j*_(*t*) can be written

$$s_{j}\left(t\right)=u_{3,j}t^{3}+u_{2,j}t^{2}+u_{1,j}t+u_{0,j}t\;j=1,2,\ldots ,n$$ (7)

The value of *s*_*j*_(*t*) at each interval endpoint is equal to the value at the sample point, i.e. *s*_*j*_ (*t*_*j*-1_) = *q*_*j*-1_, *s*_*j*_ (*t*_*j*_) = *q*_*j*_. The derivatives of *s*_*j*_(*t*) at the endpoints of each interval are continuous, i.e. *s*_*j*_′(*t*_*j*_ -0) = *s*′_*j*_ (*t*_*j*_ +0). The second-order derivatives of *s*_*j*_(*t*) at the endpoints of each interval are continuous, i.e. *s*_*j*_′′(*t*_*j*_ -0) = *s*_*j*_′′(*t*_*j*_ +0).

Noting that *s*_*j*_′′(*t*) is a first-order polynomial on the closed interval [*t*_*j*-1_, *t*_*j*_], one can assume that the values of *s*_*j*_′′(*t*) at the endpoints of the interval are known, i.e. *s*_*j*_′′(*t*_*j*-1_) = *M*_*j*-1_, *s*_*j*_′′(*t*_*j*_) = *M*_*j*_. Then

$${s^{\prime \prime }}_{j}\left(t\right)=\frac{\left(t_{j}-t\right)M_{j-1}+\left(t-t_{j-1}\right)M_{j}}{h_{j}}$$ (8)

where *h*_*j*_ = *t*_*j*_ - *t*_*j*-1_.

By computing the integral of Eq. 8, we are able to obtain a general expression for the cubic spline at any moment t in the closed interval [*t*_*j*-1_, *t*_*j*_]

$$s_{j}\left(t\right)=\frac{{\left(t_{j}-t\right)}^{3}M_{j-1}+{\left(t-t_{j-1}\right)}^{3}M_{j}+\left(t_{j}-t\right)\left(6w_{j-1}-M_{j-1}h_{j}^{2}\right)+\left(t-t_{j-1}\right)\left(6w_{j}-M_{j}h_{j}^{2}\right)}{6h_{j}}$$ (9)

From Eq. 8, we can obtain that there are (n+1) unknown variables in the whole trajectory *s*(*t*) (*M*_0_, *M*_1_, ..., *M*_*n*_). In order to find out all the parameters, it is necessary to construct independent equations for *M*_0_, *M*_1_, ..., *M*_*n*_ of independent equations

$$\mu_{j}M_{j-1}+2M_{j}+\lambda_{j}M_{j+1}=\gamma_{j}$$ (10)

where *μ*_*j*_ = *h*_*j*_/(*h*_*j*_+*h*_*j*+1_), *λ*_*j*_ *=* 1-*μ*_*j*_, *γ*_*j*_ = 6 [(*w*_*j*+1_-*w*_*j*_)/*h*_*j*+1_-(*w*_*j*_-*w*_*j-*1_)/*h*_*j*_]/(*h*_*j*_+*h*_*j*+1_).

For *n* cubic polynomials consisting of *s*(*t*), there are (*n*-1) internal knots to obtain (*n*-1) equations in Eq. 8. In order to solve for the (*n*+1) unknown variables, we still need two other constraints. Noting that the velocity at the start and end of the whole trajectory is zero, i.e. *s*_*j*_′(0) = 0, *s*_*j*_′(*t*_*n*_) = 0. This leads to (*n*+1) independent equations

$$\left[\begin{array}{cccccc} 2 & 1 & & & & \\ \mu_{1} & 2 & \lambda_{1} & & & \\ & \mu_{2} & 2 & \lambda_{2} & & \\ & & \ddots & \ddots & \ddots & \\ & & & \mu_{n-1} & 2 & \lambda_{n-1}\\ & & & & 1 & 2 \end{array}\right]\left[\begin{array}{c} M_{0}\\ M_{1}\\ M_{2}\\ \vdots \\ M_{n-1}\\ M_{n} \end{array}\right]=\left[\begin{array}{c} \gamma_{0}\\ \gamma_{1}\\ \gamma_{2}\\ \vdots \\ \gamma_{n-1}\\ \gamma_{n} \end{array}\right]$$ (11)

where *μ*_*i*_, *λ*_*i*_ are known quantities and *M*_*i*_ are unknown variables.

The solution of Eq. 11 determines the display equation for the cubic polynomial, so the time-optimal problem can be described as solving for the minimum of the sum of *h*_*j*_ within the constraints, i.e.

$$\begin{array}{l} f\left(t\right)=\sum_{j=0}^{n}t_{j}\\ s\mathrm{.t}.\;\{\begin{array}{c} \left|\max\left(s^{\prime }\left(t\right)\right)\right|\leq V_{\max}\\ \left|\max\left(s^{\prime \prime }\left(t\right)\right)\right|\leq A_{\max}\\ \left|\max\left(s^{\prime \prime \prime }\left(t\right)\right)\right|\leq J_{\max} \end{array} \end{array}$$ (12)

## Improved Sparrow Search Algorithm

### Sparrow Search Algorithm

The sparrow search algorithm models the behaviour of the sparrow as it forages for food and escapes predation. As with other heuristics, the sparrow population can be understood as a randomly generated variable. The algorithm defines the different identities of individuals in the population: producers and joiners. The process of sparrow foraging is, in fact, an algorithmic search for optimal performance. According to a certain ratio, individuals with good energy are defined as producers during each foraging session and the remaining individuals as joiners. The joiner moves closer to the producer. The fitness function measures the magnitude of the energy. In addition, a certain percentage of individuals (typically 10–20% of the entire population) are randomly selected from the entire sparrow population to sense danger. These sparrows act like variant individuals in GA, preventing the population from falling into a local optimum. Their position shifts when certain conditions are triggered. This increases the diversity of the population and improves the possibility of individuals escaping the local optimum.

In the *T*th foraging action, the producer’s position is updated as follows

$$x_{i,j}^{T+1}=\{\begin{array}{c} x_{i,j}^{T}\exp\left(-\frac{i}{\alpha \cdot iter_{\max}}\right),\;R_{2}<ST\\ x_{i,j}^{T}+Q\cdot L,\;\;\;\;\;\;R_{2}\geq ST \end{array}$$ (13)

where *T* is the current number of iterations, *i* is the sequence of individual sparrows in the population, and *x*_*i*,*j*_ denotes the coordinate position of the *jth* dimension of the current *ith* producer. Random factor *α*∈(0,1], *iter*_*max*_ represents the maximum number of iterations, *Q* is a random value that follows a normal distribution, *L* is a vector of the same dimension as the individual sparrow, random numbers *R*_2_∈[0,1], *ST*∈[0.5, 1].

*R*_2_ is a random number and *ST* is a constant in the domain of definition. *R*_2_ determines how the producer updates and this random setting allows for more variation in the producer’s position, increasing the possibility of optimization seeking. For joiners

$$x_{i,j}^{T+1}=\{\begin{array}{c} Q\exp\left(-\frac{x_{worst}-x_{i,j}^{T}}{i^{2}}\right),i>\frac{n}{2}\\ x_{p}^{T+1}+\left|x_{i,j}^{T}-x_{p}^{T+1}\right|A^{+}L,i\leq \frac{n}{2} \end{array}$$ (14)

where *x*_*worst*_ is the individual sparrow with the weakest current energy, *n* is the number of joiners, $x_{p}^{T+1}$ is the sparrow with the highest energy in Equation 10; ***A*** is a vector of the same dimension as the individual sparrow, with internal elements 1 and -1, ***A*** ^+^ = ***A*** ^T^ (***AA*** ^T^) ***A*** ^−1^.

The accessions are renewed in two ways, preserving the distribution of the sparrow population. For the sparrow responsible for early warning

$$x_{i,j}^{T+1}=\{\begin{array}{c} x_{best}^{T}+\beta \left|x_{i,j}^{T}-x_{best}\right|,\;\;f_{i}>f_{g}\\ x_{i,j}^{T}+K\left(\frac{x_{i,j}^{T}-x_{worst}}{\left(f_{i}-f_{w}\right)+\varepsilon }\right),\;f_{i}=f_{g} \end{array}$$ (15)

where *x*_*best*_ is the globally optimal individual in the current action, *f*_*i*_ is the fitness value of the individuals, and *f*_*g*_, *f*_*w*_ are the optimal fitness and the worst fitness, respectively, that the sparrow population can achieve in this action. *β*, *K* are step coefficients. *ε* is a minimal value that prevents the denominator from being zero.

The sparrow responsible for the warning is at the edge of the population when *f*_*i*_ > *f*_*g*_ and should move closer to the position where the globally optimal sparrow is located. The sparrow responsible for sensing danger chooses to update its position towards the centre of the group in order to reduce the risk of predation when *f*_*i*_ = *f*_*g*_. This setup aims to avoid too many individuals reaching a local optimum, and the algorithm stops iterating and falling into a local optimum.

### Initial Population Optimization Based on Tent Chaotic Mapping

In the original algorithm, the authors compared the performance of SSA and GSA, PSO, GWO on nineteen tested functions. SSA has a robust global search capability and yields high accuracy, outperforming other algorithms in multi-modal search problems. The distribution of the initial population is closely related to the performance of the algorithm. A uniformly distributed initial population can enrich the diversity of the population and thus improve the search efficiency of the algorithm. However, this consideration was not made in the original SSA, and the implementation of the algorithm relied on pseudo-random numbers to complete the initial population take. Tent chaotic sequences are stochastic and ensure the diversity of populations (Liu et al., 2016). Tent mapping can produce a uniformly distributed initial population, which effectively prevents individuals from approaching the local optimum too early. The expression for the Tent chaos mapping is

$$x_{i+1}=\{\begin{array}{c} 2x_{i},\;0\leq x_{i}\leq 0.5\\ 2\left(1-x_{i}\right),\;0.5<x_{i}\leq 1 \end{array}$$ (16)

In order to avoid the sequence of Tent chaotic mappings falling into small and unstable periodic points during iteration, a random variable *δ/N* is introduced into the original Tent mapping

$$x_{i+1}=\{\begin{array}{c} \frac{2x_{i}+\delta /N-x_{\min}}{x_{\max}-x_{\min}},\;\;\;0\leq x_{i}\leq 0.5\\ \frac{2\left(1-x_{i}\right)+\delta /N-x_{\min}}{x_{\max}-x_{\min}},\;0.5<x_{i}\leq 1 \end{array}$$ (17)

where the random variable *δ* ∈ [0,1], *N* is the number of elements in the individual sparrow, and *x*_min_, *x*_max_ are the smallest and largest elements in the individual sparrow.

**FIGURE 1:**
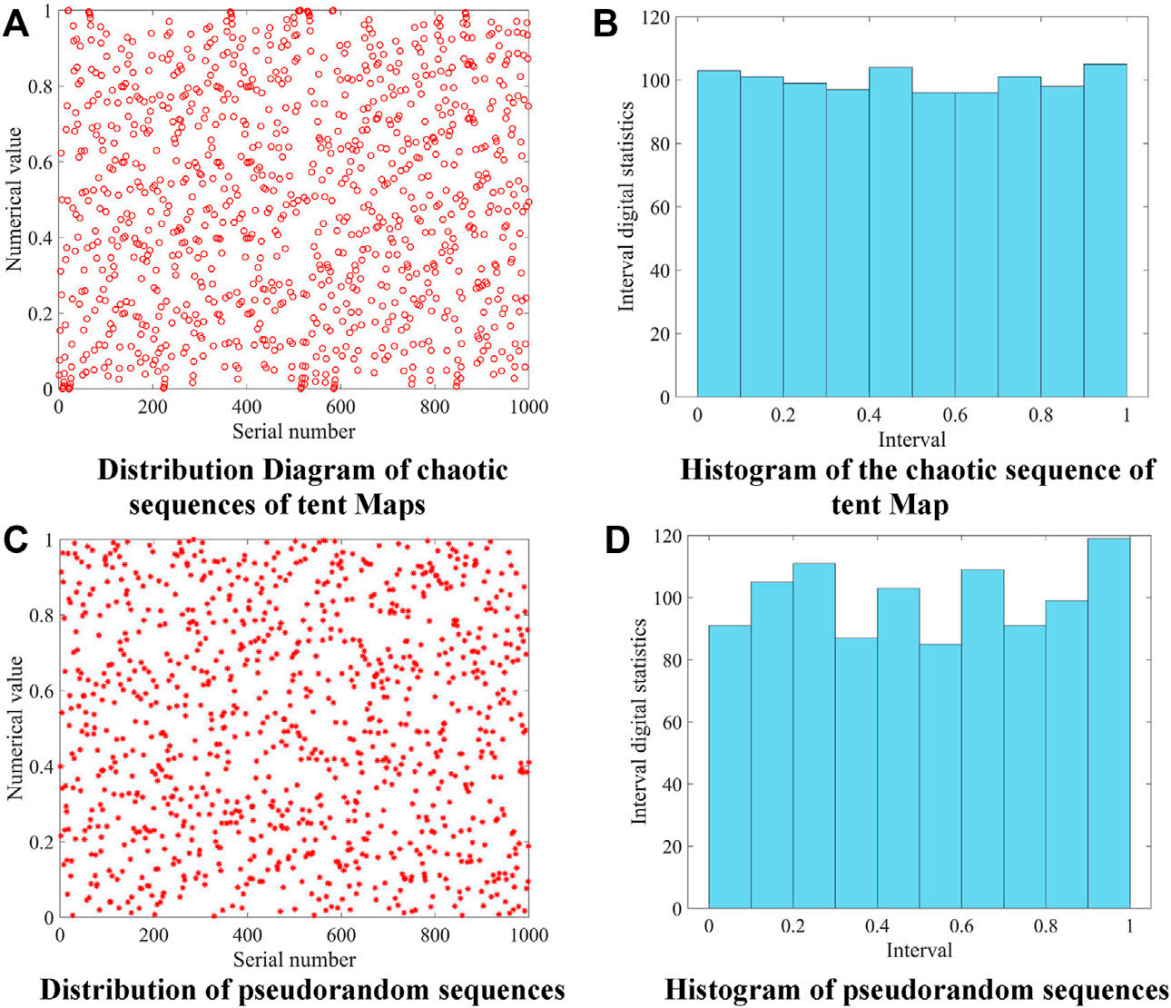
Comparison of two kinds of random number generators.

To further compare pseudo-random numbers and Tent chaotic sequences, 1,000 sets of random numbers with values in [0,1] were generated by two methods. The intervals were evenly divided into 10 parts, and the frequency of occurrence of the numbers in each interval was counted. Figures 1A,C visualize the location of the random numbers generated in the two ways, while Figures 1B,D count the number of occurrences of the random numbers in each interval. It can be seen that the improved Tent mapping produces a more uniform distribution of chaotic sequences of random numbers.

### Adaptive Step Factor

**FIGURE 2:**
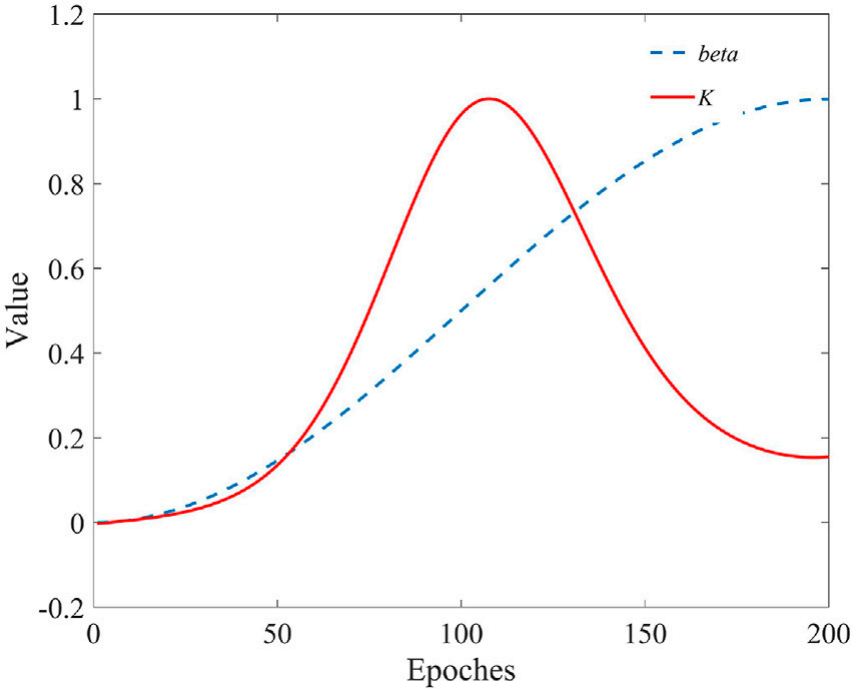
Variation of step size factors with the number of iterations.

The function of Eq. 15 is to avoid that too many individuals reach the optimum, and the algorithm stops iterating, thus falling into a local optimum. However, its effect is weakened because the step factor in Eq. 15 is random. The sparrows should approach the individual with the best position more quickly when *f*_*i*_ > *f*_*g*_, thus achieving accelerated convergence. The cosine annealing algorithm is widely used in deep learning and has excellent conditioning effects. The cosine annealing algorithm was used to adjust the step size factor *β*. In early searches, sparrow populations were more widely distributed, global search capacity was more robust and local search capacity needed to be increased. Using Eq. 19, the value of *K* is adjusted to increase in the early period and decrease rapidly in the later period. Such an adaptation ensures that the algorithm maintains a robust global search capability in the early stages, and accelerates convergence in the later stages. The variation of the two step factors with the number of iterations is shown in Figure 2. Equations 18 and 19 are defined as adaptive step factors, which we refer to uniformly as ADF in the rest of the section

$$\beta =\beta_{\min}+0.5\left(\beta_{\max}-\beta_{\min}\right)\left(1-\cos\left(iter_{cur}/iter_{\max}\right)\right)$$ (18)

$$K=\left(iter_{cur}/iter_{\max}\right)\exp\left(\sin{\left(\frac{iter_{cur}}{iter_{\max}}\pi \right)}^{2}-K_{\min}\right)/K_{\max}$$ (19)

where *β*_min_ and *β*_max_ are the minimum and maximum values of *β*. *K*_min_ and *K*_max_ are the lower and upper limits of the value of *K*. *iter*_*cur*_ and *iter*_*max*_ are the current number of iterations and the total number of iterations respectively.

**FIGURE 3:**
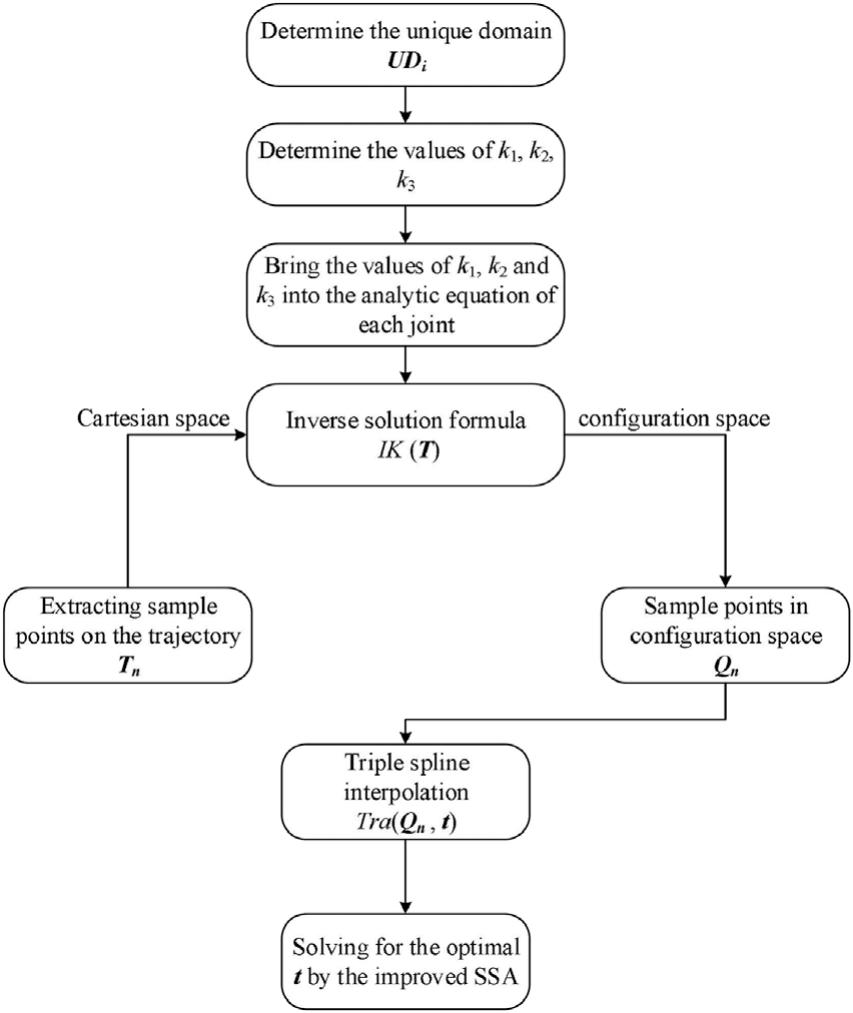
Time-optimal algorithm flow for trajectories.

Equations 13–15, derived from the original work of SSA (Xue and Shen, 2020), specify how the sparrow is updated and construct the basic flow of the algorithm. Based on the idealization and feasibility of the above model, the basic steps of the improved SSA can be summarized in the pseudo-code shown in Algorithm 1. The algorithm flow for solving the time-optimal trajectory can be obtained from the above work, as shown in Figure 3. ***UD***_***i***_ in the figure is a shorthand for the unique domain, *i* = 1,2,3,...,8.

Algorithm 1 The framework of the improved SSA

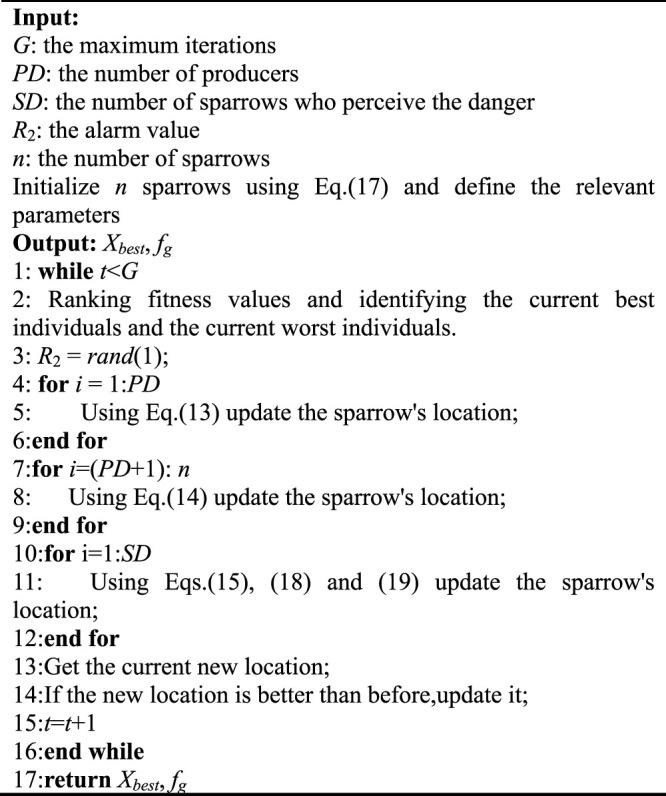

## Experiments Based on UR5 Cobot

**FIGURE 4:**
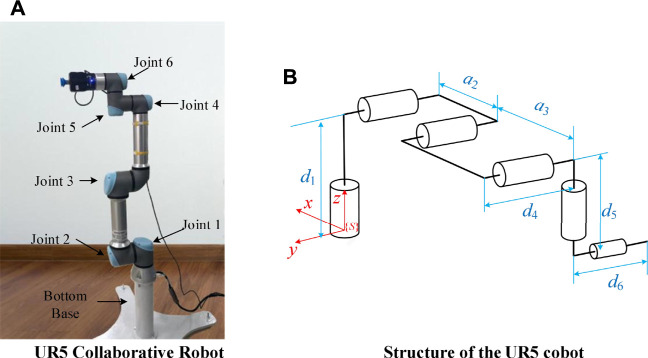
The solid and structure of the UR5 robot.

Experiments were carried out on the UR5 cobot platform in order to verify the validity of the work presented earlier. The solid and structure of the UR5 robot are shown in Figures 4A,B, and the DH parameters are shown in Table 1.

**FIGURE 5:**
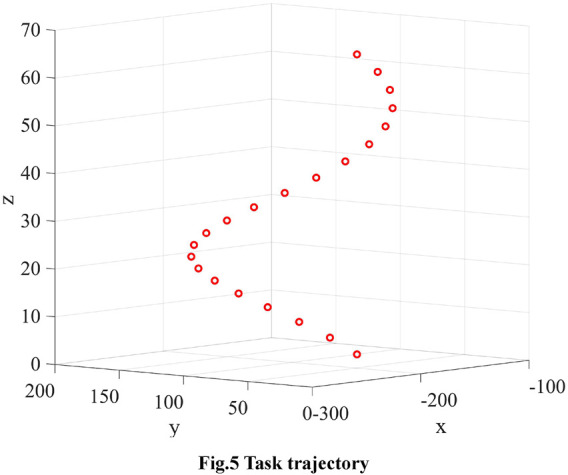
Task trajectory.

The task trajectory is a spiral line with a circle centre position of (-200,100)mm, a radius of 60 mm and a pitch of 10 mm in a Cartesian coordinate system. The trajectory of the helix in the Cartesian system is shown in Figure 5 and the coordinates of the sampling points are shown in Table 2.

**TABLE 2:** Positions of every sample points.

No	*x*/mm	*y*/mm	*z*/mm
1	−140.000	100.000	0.000
2	−142.937	118.541	3.142
3	−151.459	135.267	6.283
4	−164.733	148.541	9.425
5	−181.459	157.063	12.566
...	...		
19	−151.459	64.733	56.549
20	−142.937	81.459	59.690

The efficiency of the inverse solution calculation during robot motion is important and affects the efficiency of the overall motion. A single calculation can derive the joint angles for all sample points by Eq. 4 in Section 3. The joint angles corresponding to each sample point are obtained by inverse kinematics as shown in Table 3. Since the pose did not change during the motion along the trajectory, no motion occurred for joint 6, no trajectory planning was required for joint 6.

**TABLE 3:** The corresponding angle of the sampling point.

No	*θ*_1_/rad	*θ*_2_/rad	*θ*_3_/rad	*θ*_4_/rad	*θ*_5_/rad	*θ*_6_/rad
1	−1.5066	2.2871	−2.4364	−2.9923	1.5066	3.1416
2	−1.4931	2.2125	−2.4216	−2.9325	1.4931	3.1416
3	−1.4451	2.1280	−2.3985	−2.8711	1.4451	3.1416
4	−1.3785	2.0459	−2.3707	−2.8168	1.3785	3.1416
5	−1.3023	1.9726	−2.3418	−2.7724	1.3023	3.1416
...	...					
19	−1.34683	2.2836	−2.5835	−2.8417	1.3468	3.1416
20	−1.46255	2.2808	−2.5912	−2.8312	1.4626	3.1416

**FIGURE 6:**
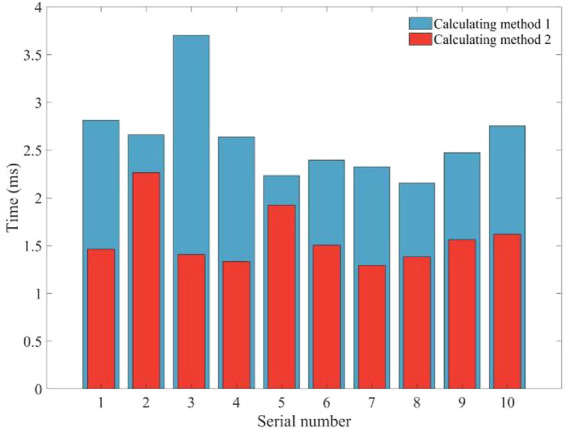
Time results for ten calculations of the two inverse solution schemes.

Compared to the method of calculating eight sets of inverse solutions and then filtering them by the principle of minimum displacement (known as calculation method 1), the method of joint space division (known as calculation method 2) is more time-efficient. The two inverse solution calculation methods were run ten times in Matlab 2019b, and the calculation time results are shown in Figure 6. The average time for ten runs of the two methods was 2.6149 and 1.5752 ms respectively, with the joint space division based method able to save 39.76% of the time. This proportion increases linearly with the number of sampling points.

**FIGURE 7:**
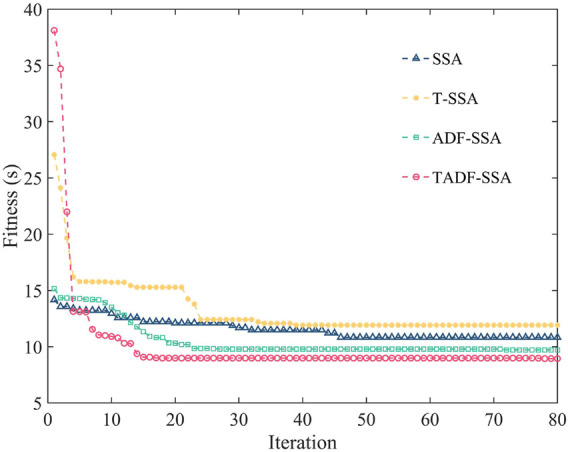
Comparison of the results of the improved SSA with the original algorithm.

The maximum velocity and maximum acceleration constraints for each joint are shown in Table 4. We did controlled experiments in the same environment to further investigate the advantages of the two improvements to SSA in this paper. The maximum number of iterations is 80, the population size is 100, and the number of discoverers is 20% of the entire population. The sparrows responsible for scouting were 10% of the population, and *ST* = 0.5 in Eq. 13. To compare the effects of the two improvements on SSA, the same trajectory is optimized with the same initial parameters. SSA combined with Tent chaos mapping is referred to as T-SSA. SSA combined with an adaptive step factor is referred to as ADF-SSA. The SSA with both improvements is called TADF-SSA. To verify the stability of the algorithms, the variations of the fitness functions of the four algorithms are run ten times separately at the same hardware level. The optimal performance of each algorithm is shown in Figure 7. The results of the ten runs are averaged and recorded in Table 5.

**TABLE 4:** Constraint conditions of each joint.

	Joint 1	Joint 2	Joint 3	Joint 4	Joint 5
*V*_max_ (rad/s)	1.7453	1.6581	1.7453	2.6180	2.2689
*A*_max_ (rad/s^2^)	0.7854	0.6981	1.3090	1.2217	1.5708
*J*_max_ (rad/s^3^)	1.0472	1.0472	0.9599	1.2217	1.3090

**TABLE 5:** Average of convergence after 10 runs of the four algorithms at the terminal of Intel(R) i7-9750H CUP@ 2.60 GHz.

	SSA	T-SSA	ADF-SSA	TADF-SSA
Iterations at convergence	47.8	34.3	24.3	18.2
Fitness at convergence (s)	10.87	12.14	9.91	9.04

From Figure 7, we can see that the best convergence result for SSA is 10.83s after 46 iterations. T-SSA converges to a minimum value of 12.07s after 34 iterations. ADF-SSA converges to a minimum value of 9.85s after 23 iterations. TADF-SSA converges to a minimum value of 8.99s after 18 iterations. As can be seen from Table 5, the average results of the ten runs float around the best results, and the algorithm’s performance is stable. The results of T-SSA and SSA shows that using Tent chaotic mapping alone does not improve the algorithm significantly. This is since the Tent chaotic mapping reduces the effect of population precocity, while the search capability of the SSA algorithm itself causes this result. The results of ADF-SSA and SSA shows that the adaptive step factor (ADF) can improve the global search capability and efficiency of the algorithm. TADF-SSA performed best in all results, which demonstrates the effectiveness of the improvements to the SSA algorithm in this paper.

**FIGURE 8:**
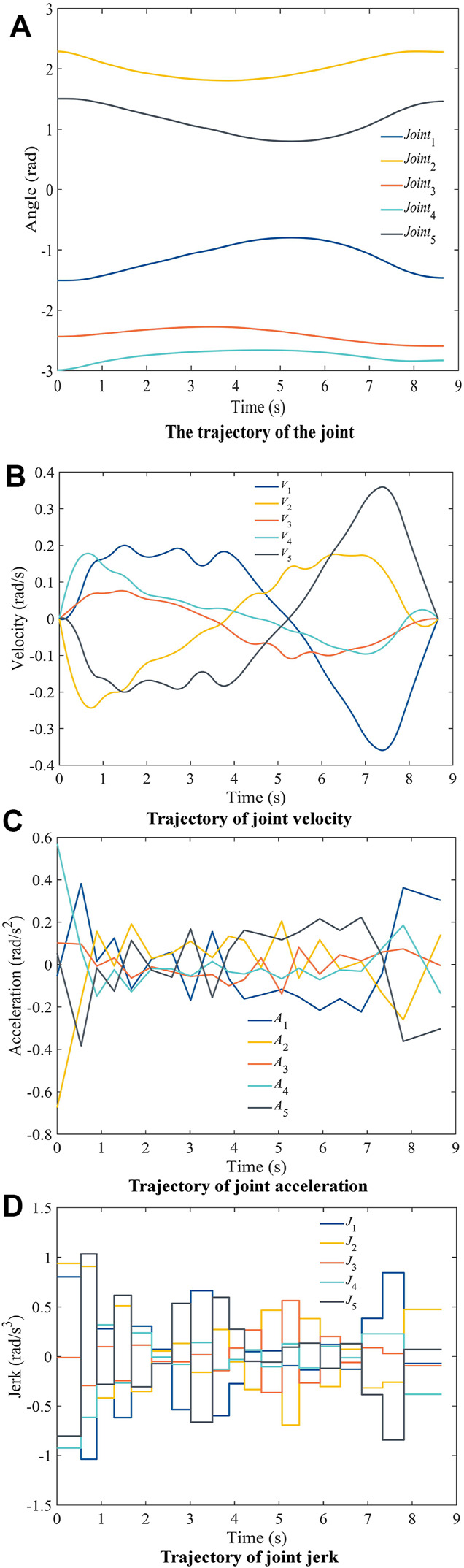
Results of time-optimal trajectory planning for angle, velocity, acceleration and jerk.

The initial time was set to 50s, and the trajectory obtained after time optimization by the improved SSA is shown in Figure 8. The movement time of the whole trajectory is 8.6618s (the average of 5 runs is taken as the final time to ensure the algorithm’s stability), which is 82.68% shorter. The interpolation time for each segment is shown in Table 6. The trajectory of each joint's angle (as shown in Figure 8A) and velocity (as shown in Figure 8B) changes is very smooth, and the maximum value is less than the constraint. The acceleration profile is continuous, as shown in Figure 8C, and the maximum value of the angular acceleration of each joint is less than the constraint. The jerk of each joint angle is also much smaller than the constraint, as shown in Figure 8D, which ensures that the motor is protected from large shocks.

**TABLE 6:** Interpolation time for each segment of the trajectory.

	*h*_1_	*h*_2_	*h*_3_	*h*_4_	*h*_5_	*h*_6_	*h*_7_	*h*_8_	*h*_9_	*h*_10_	*h*_11_	*h*_12_	*h*_13_	*h*_14_	*h*_15_	*h*_16_	*h*_17_	*h*_18_	*h*_19_
Time/s	0.5439	0.3541	0.3914	0.3878	0.4609	0.4540	0.4237	0.4882	0.3734	0.3460	0.3829	0.4649	0.3870	0.4704	0.4555	0.4819	0.4686	0.4803	0.8469

**FIGURE 9:**
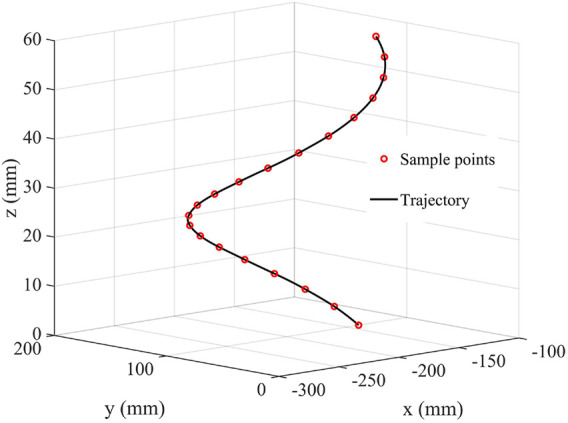
The trajectory of EE.

The trajectory of the obtained joint angles is mapped by forward kinematics to the trajectory of EE in a Cartesian coordinate system as shown in Figure 9. It can be seen that the trajectory of EE in Cartesian space is very smooth and passes through each sampling point. The result is consistent with engineering applications and practical requirements.

**FIGURE 10:**
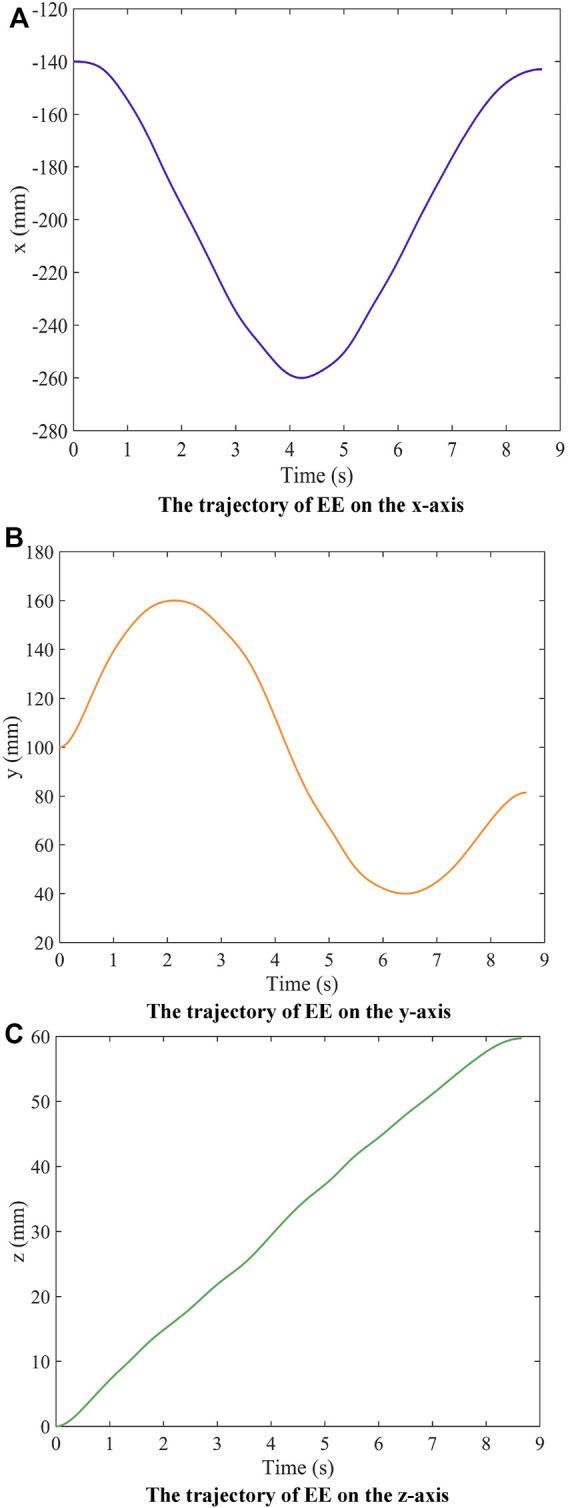
The trajectory of EE on the three axes of motion.

The variation of the components of the end trajectory mapped to the three axes in Cartesian space is shown in Figures 10A‐C correspond to the trajectories of axes x, y, and z, respectively). The trajectories are smooth and continuous in all three coordinates, which indicates that the EE is moving smooth and stable in the set trajectory.

## Conclusion

An optimal time trajectory planning method is proposed in terms of both inverse kinematic solution and time optimization. Trajectory planning is accomplished in the joint space through cubic spline interpolation, and the joint space is refined to improve the inverse solution calculation time. The sparrow search algorithm is optimized for the initial population generation method and step factor update. An improved tent chaotic mapping improves the rationality of the initial population distribution. The global search capability of the algorithm is improved by combining the step size factor of the cosine annealing algorithm. A time-optimal trajectory was obtained by an improved sparrow search algorithm. Simulation experiments were carried out on the UR5 collaborative robot, and the results showed that the time of the obtained trajectory was considerably optimized while satisfying the constraints. The feasibility and effectiveness of the algorithm were verified.

The method proposed in this paper for calculating time-optimal trajectories is applicable to tandem robots of any configuration. The method of calculating the inverse solution when planning trajectories can improve the efficiency of robot motion, but this method relies on the robot’s structure and has not been generalized to robots of general configuration, which requires further theoretical study.

## Data Availability

The original contributions presented in the study are included in the article/Supplementary Material, further inquiries can be directed to the corresponding authors.
